# Recombinant cell-penetrating trichosanthin synergizes anti-PD-1 therapy in colorectal tumor

**DOI:** 10.7150/ijbs.81052

**Published:** 2023-03-05

**Authors:** Meng Zhang, Ergang Liu, Guihua Chen, Yisi Tang, Yuefei Fang, Yang He, Pengfei Zhao, Caihong Zheng, Bahtiyor Muhitdinov, Yongzhuo Huang

**Affiliations:** 1 Department of Pharmacy, Women's Hospital, Zhejiang University School of Medicine, Hangzhou 310006, China; 2 State Key Laboratory of Drug Research, Shanghai Institute of Materia Medica, Chinese Academy of Sciences, Shanghai 201203, China; 3 Zhongshan Institute for Drug Discovery, Shanghai Institute of Materia Medica, Chinese Academy of Sciences, Zhongshan 528437, China; 4 Artemisinin Research Center, Guangzhou University of Chinese Medicine, Guangzhou 510450, China; 5 Department of Pharmacy, the First Affiliated Hospital of Zhengzhou University, Zhengzhou 450052, China; 6 Clinical Pharmacology Center, the Second Affiliated Hospital, Zhejiang University School of Medicine, Hangzhou 310009, China; 7 Institute of Bioorganic Chemistry, Uzbekistan Academy of Sciences, 83, M. Ulughbek Street, Tashkent 100125, Uzbekistan; 8 NMPA Key Laboratory for Quality Research and Evaluation of Pharmaceutical Excipients, Shanghai 201203, China; 9 School of pharmaceutical sciences, Southern Medical University,1838 Guangzhou Avenue North, Guangzhou 510515, China

**Keywords:** Trichosanthin, cell-penetrating peptide, anti-PD-1 therapy, tumor immune microenvironment, colorectal cancer

## Abstract

Alleviating immunosuppression of the tumor microenvironment is an important strategy to improve immune checkpoint therapy. It is an urgent but unmet need to develop adjuvant therapeutics for assisting the mainstay immunotherapies. Trichosanthin is an approved gynecology drug in China and its immunomodulatory effects have drawn much attention as an old drug for new applications in cancer. In this work, a recombinant cell-penetrating trichosanthin (rTCS-LMWP) was prepared via genetic fusion of a cell-penetrating peptide sequence (LMWP) to trichosanthin aiming to overcome the intratumoral penetration and intracellular delivery challenges. The potential of trichosanthin as an adjuvant therapy was explored, including its effects on tumor cells, antigen-presenting cells, tumor immune microenvironment, and the synergistic effect in combination with anti-PD-1. The results revealed that rTCS-LMWP can stimulate the maturation of dendritic cells via activating the STING-TBK1-IRF3 pathway, repolarize the protumor M2-type macrophages, and upregulate the pro-inflammatory cytokine expression. Moreover, rTCS-LMWP can enhance anti-PD-1 therapeutic efficacy in a CT26-bearing mouse model. The synergistic effect involved the induction of immunogenic cell death in the tumors, the proliferation and functionalization of cytotoxic T cells, and the suppression of the immunosuppressive regulatory T cells. These findings indicate that trichosanthin can be developed as an immunomodulator to facilitate cancer immunotherapy.

## Introduction

Colorectal cancer (CRC) is the second cause of cancer-associated death with a mortality rate of 9.4% 1. Early screening has improved the 5-year survival. However, 20% of newly diagnosed CRC patients have metastatic disease, and 25% of other-stage CRC patients will develop metastatic disease 2, 3. And metastatic CRC patients have a poor prognosis with a 5-year survival rate of less than 20% 3, 4. Developing an effective treatment for CRC patients is an urgent need. Cancer immunotherapy, as a new treatment modality to drive the immune system to recognize and annihilate cancer cells, has been changing the treatment paradigm of CRC 5, 6.

Immune checkpoints are normally a self-protection mechanism to prevent excessive immune responses in the healthy body but are responsible for tumor immune escape. Immune checkpoint inhibitors (ICIs) can restore the effector cytotoxic T cells to eradicate tumor cells by releasing the brake of immune checkpoints on the immune system 7. PD-1 antibodies (pembrolizumab and nivolumab) and the combination of nivolumab and ipilimumab (CTLA-4 antibody) have been approved for the treatment of refractory CRC patients who have mismatch-repair-deficient or high microsatellite instability (dMMR-MSI-H) 5. However, the overall clinical benefit of anti-PD-1 is largely limited by the tumor immunosuppressive microenvironment (TIME) 8. The majority (around 85%) of CRC patients are immunologically cold and characterized by mismatch-repair-proficient or microsatellite instability-low (pMMR-MSI-L) 9. The pMMR-MSI-L tumors do not generate immunostimulatory neoantigen and sufficient T cell infiltration, resulting in ineffective responses to immunotherapy 9. Ameliorating the tumor immunosuppressive microenvironment is an effective strategy to improve the response rate of ICIs; for example, inducing immunogenic cell death (ICD) of tumor cells and increasing tumor-infiltrating lymphocytes (TILs) can improve the anti-PD-1/PD-L1 therapeutic efficacy 10-12.

Trichosanthin (TCS), from the root of Trichosanthes *kirilowii Maxim.*, is a type I ribosome-inactivating protein and has been clinically used for hydatidiform mole, extrauterine pregnancy, stillbirth, and abortion in China for decades 13. Notably, TCS can effectively inhibit tumors like fibrosarcoma 14 and glioma 15, and restore the sensitivity of tumors to chemotherapeutics 16, 17. In our lab, we previously revealed that TCS induced anticancer immunity, such as increasing the intratumoral infiltration of CD8^+^ T cells and stimulating the dendritic cells, thus serving as a vaccination adjuvant 18, 19. It suggested the potential of TCS as an immunostimulant to assist immune checkpoint inhibition therapy.

In this work, the effect of TCS assisting anti-PD-1 therapy was investigated in a colon tumor model. It is noteworthy that TCS is a macromolecule with poor cell permeability, thus yielding inefficient intratumoral diffusion and intracellular uptake. To address this delivery issue, a recombinant cell-penetrating TCS (termed rTCS-LMWP) was prepared by site-specific fusion with a cell-penetrating peptide (low-molecular-weight protamine, LMWP). LMWP is a shortened sequence derived from protamine, an FDA-approved drug, unlike the common TAT which is derived from a viral protein of HIV. Compared to TAT, LMWP has a similar arginine-rich structure and an equivalent capacity for intracellular delivery of its cargo 20. Importantly, LMWP is safer than protamine, and the biosafety profile has been confirmed in different experimental animal models; for example, LMWP is not antigenic 21, and elicits only minor complement activation and non-detectable hypotensive responses in dogs 22. Moreover, LMWP can be produced in large quantities by enzymolysis of native protamine 23, 24. Therefore, LMWP was selected to assist TCS delivery.

## Materials and Methods

### Materials

The original TCS plasmid was kindly provided by Prof. Pang-Chui Shaw at the Chinese University of Hong Kong. The pTXB vector containing Intein-mediated purification with affinity chitin-binding domain (CBD) was obtained from New England Biolabs (Ipswich, USA). L-cysteine, ampicillin, isopropyl beta-D-1-thiogalactopyranoside (IPTG), and 5-FITC were purchased from Meilun Biotechnology (Dalian, China). Lipopolysaccharide (LPS) was obtained from Sigma-Aldrich (St. Louis, USA). The recombinant murine granulocyte-macrophage colony-stimulating factor (mGM-CSF), interleukin-4 (mIL-4), interferon-gamma (IFN-γ), and macrophage colony-stimulating factor (mM-CSF) were purchased from PeproTech (Cranbury, USA). The enhanced BCA protein assay kit, secondary antibodies, red cell lysis, RIPA lysis, and DAPI were purchased from the Beyotime Institute of Biotechnology (Shanghai, China). Fetal bovine serum (FBS), RPMI-1640 medium, and Dulbecco's Modified Eagle's Medium (DMEM, Gibco) were provided by Thermo Fisher (Waltham, USA). The rabbit monoclonal anti-IRF3 (ab68481) and anti-TMEM173 (ab288157) antibodies were purchased from Abcam (Cambridge, UK). The flow cytometry antibodies including PerCP anti-mouse CD3 (Cas, 100326), FITC anti-mouse CD4 (Cas, 100509), PE anti-mouse CD8 (Cas, 100707), Brilliant Violet 510™ anti-IFN-γ (Cas, 505842), Alexa Fluor® 647 anti-FoxP3 (Cas, 126408), FITC anti-CD11c (Cas, 117306), PE anti-CD80 (Cas, 104708), PE/Cyanine7 anti-CD86 (Cas, 105013), FITC anti-F4/80 (Cas, 123107), APC anti-CD206 (Cas, 141707), and Alexa Fluor® 594 anti-CD169 (Cas, 142416) were obtained from BioLegend (California, USA). The flow cytometry antibodies including eFluor™ 660 anti-Ki67 (Cas, 50-5698-82), PE/Cyanine5.5 anti-Granzyme B (Cas, 35-8898-82), and APC anti-MHC-II (Cas, 17-5320-82) were from eBioscience (California, USA). The ELISA kits including IL-1β, IL-2, IL-6, TNF-α, and IFN-γ were purchased from Dakewe Biotech. (Shenzhen, China). The TRIeasy™ Total RNA extraction reagent, cDNA synthesis SuperMix kit, and qPCR SYBR^®^ Green Master Mix were provided by Yeasen Biotech. (Shanghai, China). The primers of IL-1β, IL-6, IL-10, IL-12, IL-23, TBK1, IRF3, IP-10, TNF-α, and iNOS were obtained from Generay Biotech (Shanghai, China) (**Table S1**). All other reagents were of analytical grade and provided by Sinopharm Chemical Reagent (Shanghai, China).

### Cells

The CT26 murine colon cancer cells, bEnd3 mouse brain microvascular endothelial cells, and human umbilical vein endothelial cells (HUVEC) were provided by the National Collection of Authenticated Cell Cultures (Shanghai, China). The CT26 cells were cultured in RPMI-1640 supplementary with high glucose and 10% FBS. The bEnd3 and HUVEC cells were cultured in DMEM supplementary with 10% FBS. The cells were incubated in an incubator at 37 °C and 5% CO_2_.

The bone marrow-derived dendritic cells (BMDCs) were induced from the bone marrow mononuclear cells derived from BALB/c mice using mGM-CSF (20 ng/ml) and mIL-4 (10 ng/ml), as described in a previous report 18. The loosely attached immature DCs were collected for the subsequent experiments.

The bone marrow-derived macrophages (BMDMs) were induced from the bone marrow mononuclear cells derived from BALB/c mice using mM-CSF (20 ng/ml) under 37 °C and 5% CO_2_ for 4-5 days. The fresh medium containing LPS (100 ng/ml) and IFN-γ (20 ng/ml) was utilized to further induce the M1 phenotype for 48 h, whereas mIL-4 (40 ng/ml) was used for M2 polarization. The polarized M1 and M2 macrophages were used for the following experiment.

### Animals

The female BALB/c mice (18-20 g) were provided by Shanghai Laboratory Animal Center Co., Ltd. (Shanghai, China), and housed at the specific-pathogen free (SPF) care facility with sterilized food pellets and water under a 12 h light/dark cycle. All animal experiments were carried out according to the protocols approved by the Institutional Animal Care and Use Committee, Shanghai Institute of Materia Medica, Chinese Academy of Sciences.

### Preparation and characterization of rTCS and rTCS-LMWP

The plasmid DNA encoding rTCS-LMWP was constructed by adding the LMWP-encoding sequence to the 3'-end of the rTCS-encoding gene using a standard PCR method. The sequence of rTCS or rTCS-LMWP was subcloned to the Ndel and Xhol site of the protein-expressing vector pTXB1 containing the intein-mediated purification with affinity chitin-binding domain (CBD) to prepare the recombinant plasmids. The recombinant plasmids were transformed into *E. coli* BL21 (New Cell & Molecular Biotech., Suzhou, China). The bacteria were cultured in the LB medium supplemented with 100 μg/ml ampicillin at 37 °C and 250 rpm. When OD_600 nm_ reached around 0.6, IPTG (final concentration, 0.3 mM) was added to induce the protein expression at 25 °C and 150 rpm for 16 h. The bacteria were collected via 9, 000 rpm centrifugation for 3 min and resuspended with binding buffer (20 mM HEPES, 500 mM NaCl, 1 mM EDTA, pH 8.5). The resuspension was sonicated using an ultrasonic homogenizer (Scientz Biological Technology, Ningbo, China). The supernatant was harvested via centrifugation at 12, 000 rpm and 4 °C for 40 min and then loaded onto the pre-equilibrated chitin column. The binding proteins were cleaved with the L-cysteine-containing binding buffer by overnight incubation at 4 °C. The target protein was collected and concentrated by an ultrafiltration centrifuge tube (MWCO, 10 kDa) at 4 °C and 4, 000 rpm, and further purified using fast protein liquid chromatography (ӒKTApurifier 10, GE Healthcare, Fairfield, USA) equipped with a desalting column (GE Healthcare). The purified protein was quantified using an enhanced BCA protein assay kit and characterized by SDS-PAGE electrophoresis and MALDI-TOF-MS (MALDI TOF/TOF 5800 analyzer, AB Sciex, Framingham, USA).

### Cellular uptake of rTCS and rTCS-LMWP

rTCS and rTCS-LMWP were labeled with 5-FITC via Michael addition reaction between the -NH_2_ of protein and -N=C=S of FITC. The cellular uptake of FITC-labeled rTCS and rTCS-LMWP in CT26 cells, BMDCs, and M2-type BMDMs was detected via an inverted fluorescence microscope and flow cytometry.

The CT26 cells, BMDCs, or BMDMs were seeded into the 24-well plates at a density of 5 × 10^4^ cells/well and cultured for 24 h. The cells were treated with equivalent FITC-labeled rTCS or rTCS-LMWP for 4 h. Afterward, the cells were washed with PBS three times, fixed with 4% paraformaldehyde for 20 min, and stained with DAPI for 15 min. Fluorescent images were captured by the inverted fluorescence microscope (CARL ZEISS, Oberkochen, Germany).

The CT26 cells, BMDCs, or BMDMs were seeded into the 24-well plates at a density of 5 × 10^4^ cells/well and cultured for 24 h. The cells were treated with equivalent FITC-labeled rTCS or rTCS-LMWP for 4 h. The cells were collected and washed with PBS three times. The cellular uptake efficiency was detected using flow cytometry (Agilent NovoCyte, Palo Alto, USA).

### Tumor spheroid penetration of rTCS and rTCS-LMWP

rTCS and rTCS-LMWP were labeled with Cy5-NHS. The CT26 cells were seeded into the 96-well plates pre-coated with 1% (w/v) agarose gel at a density of 5 × 10^3^ cells/well and cultured for 5 days. The thus-formed tumor spheroids were treated with Cy5-labeled rTCS or rTCS-LMWP for 5 h. After being rinsed with PBS three times, the tumor spheroids were placed in confocal chambers and subjected to a confocal laser scanning microscope (CLSM) (TCS-SP8, Leica, Germany).

### Cell viability assay

The cells of BMDCs, M2-BMDMs, bEnd3, and HUVEC were seeded into the 96-well plates at a density of 5 × 10^3^ cells/well for 24 h. The cells were treated with different concentrations of rTCS and rTCS-LMWP for 24 h. A standard MTT assay was carried out and absorbance at 490 nm was determined using a microplate reader (Thermo Scientific, Varioskan® Flash, USA).

### Immunogenic cell death induction assay

The CT26 tumor cells were seeded into the 24-well plates at a density of 5 × 10^4^ cells per well for 24 h. The cells were treated with different concentrations of rTCS and rTCS-LMWP for 8 h. The treated cells and the supernatant were harvested separately. The collected cells were washed with PBS three times and stained with the Alexa Fluor^®^ 647-anti-Calreticulin at 4 °C for 30 min, followed by flow cytometry analysis (BD FACS Calibur, Franklin Lakes, USA). ATP and HMGB1 levels of the supernatant from CT26 cells after treatments were detected by using the ATP release assay kit and HMGB1 ELISA kit.

The tumor cells were seeded onto the round coverslips placed in the 24-well plates at a density of 5 × 10^4^ cells per well for 24 h. The cells were treated with rTCS or rTCS-LMWP at a final concentration of 5 μM for 8 h. The cell-attached coverslips were washed with PBS three times and fixed with 4% paraformaldehyde for 10 min, followed by PBS washing and Alexa Fluor 488-anti-Calreticulin staining. The cells were then stained with DAPI and detected under a confocal laser scanning microscope (CLSM) (TCS-SP8, Leica, Germany).

### Stimulation of BMDCs and BMDMs

The BMDCs were seeded into the 12-well plates at a density of 1 × 10^5^ cells per well and incubated with rTCS or rTCS-LMWP (5 μM) for 24 h. The cells were then collected and washed three times with PBS. The STING pathway and DC maturation-associated markers were detected via a standard RT-qPCR, flow cytometry, or western blotting method.

For RT-qPCR, the cells were treated with the total RNA extraction solution. The extracted RNAs were then used as templates to prepare cDNAs by using a cDNA synthesis SuperMix kit. cDNA was amplified with the qPCR SYBR^®^ Green Master Mix and the primers, and the mRNA levels of the target molecules were calculated with GADPH as the internal reference.

For flow cytometry, the cells were stained with anti-CD11c, anti-CD80, anti-CD86, and anti-MHC-II. The stained cells were detected by flow cytometry (Calibur, BD Pharmingen, USA).

For western blotting, the cells were lysed with the RIPA lysate containing cocktail protease inhibitors. The supernatant was collected by centrifugation, and the protein concentration was determined by the enhanced BCA protein assay kit. The western blotting procedure was carried out with staining with anti-IRF3, anti-TMEM173, and anti-GAPDH antibodies, and the images were taken using a gel imaging system (Biorad, USA).

The polarized M2 macrophages in the 12-well plates were treated with rTCS or rTCS-LMWP (5 μM) for 24 h. The cultured medium was discarded and the cells were washed with PBS three times, followed by a standard RT-qPCR assay to detect pro-inflammatory and anti-inflammatory cytokines.

### *In vivo* therapeutic effect

The tumor model was constructed by subcutaneously injecting CT26 tumor cells into the back of the right forelimb of the mice. On day 6 after inoculation, the mice were randomly divided into four groups, as untreated group (n = 5), rTCS-LMWP group (n = 5), anti-PD-1 group (n = 5), and rTCS-LMWP + anti-PD-1 group (n = 6). rTCS-LMWP was peritumorally injected once every two days at a dose of 250 μg/kg three times, and anti-PD-1 was intraperitoneally injected once every two days at a dose of 10 mg/kg five times. The tumor sizes were determined by detecting the tumor length (L, mm) and width (W, mm) via a vernier caliper every two days. The tumor volume (V, mm^3^) was calculated as the formula:

V = L × W^2^/2

When the tumor volume reached about 2000 mm^3^, the mice were humanely sacrificed and the tumors were harvested for further assay.

### *In vivo* remodeling of the TIME

The CT26 tumor-bearing mice were randomly divided into four groups (n = 3) on the 6^th^ day of inoculation and started treatment as described above. On the 7^th^ day after the last treatment, the tumors were harvested for flow cytometry, ELISA, and immunofluorescence detection, and the immune responses in the TME were analyzed.

For flow cytometry, the tumor tissues were prepared into single-cell suspensions. The cells were stained with the flow cytometry antibodies including anti-CD3, anti-CD4, anti-CD8, anti-IFN-γ, anti-Granzyme B, anti-FoxP3, anti-F4/80, anti-CD86, anti-CD206, and anti-CD169 according to a standard protocol, and then subjected to flow cytometry.

For ELISA, the tumor tissues were mechanically crushed and then lysed with the RIPA lysate solution containing cocktail protease inhibitors. The supernatants were used for detecting the cytokine levels using ELISA kits after adjusting the same protein concentrations.

For immunofluorescence staining, the tumor sections were pretreated with 5% BSA containing 0.03% triton-X 100, followed by incubation with anti-CD206, anti-PD-L1, anti-CD8, anti-Calreticulin, and anti-HMGB1 antibodies for overnight at 4 °C. The sections were incubated with the Alexa Fluor 488 or Cy3-labeled secondary antibodies at room temperature for 1 h and then stained with DAPI for 20 min. The treated slides were observed and recorded by CLSM (TCS-SP8, Leica, Germany).

### Preliminary safety evaluation of rTCS-LMWP

The BALB/c mice were randomly divided into three groups, as untreated, CpG ODNs (ODN 1826), and rTCS-LMWP. CpG ODNs (250 μg/kg) and rTCS-LMWP (250 μg/kg) were subcutaneously continually injected three times once every two days. The body weight was measured before injection. At 6 h after the last dosing, the mice were euthanized and the blood, skin of the injection site, and major organs were collected for serum cytokine detection, skin H&E staining, and organ coefficient determination, respectively.

### Statistics

All the data were shown as mean ± standard deviation (SD) (n ≥ 3). The statistical analysis was performed by t-test and one-way ANOVA. Statistically, the significant difference was defined as *p < 0.05; **p < 0.01; ***p < 0.001; and ****p < 0.0001; ns, not significant.

## Results

### Preparation and characterization of rTCS and rTCS-LMWP

**Figure 1:**
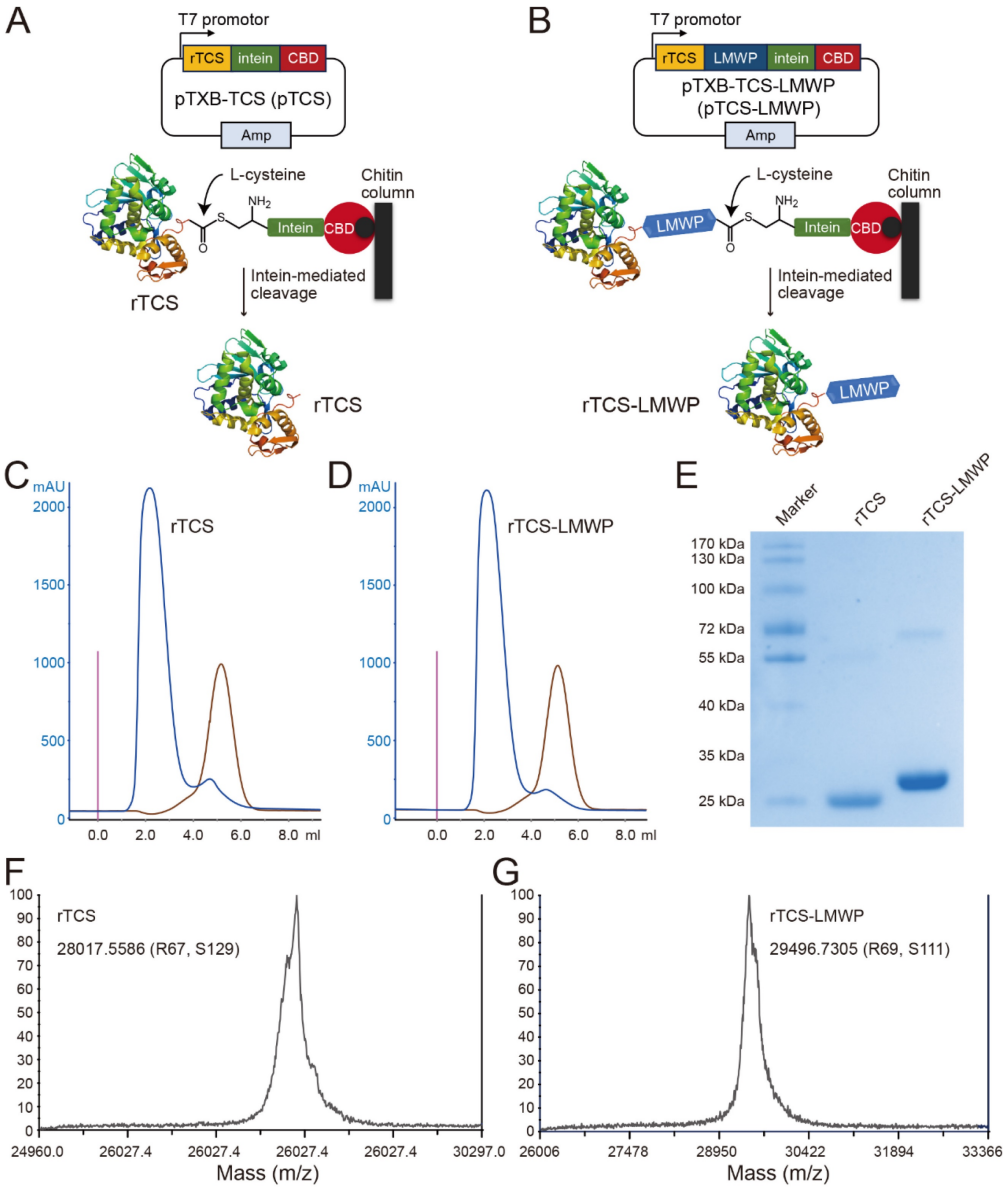
Preparation and characterization of rTCS and rTCS-LMWP. Scheme of intein-mediated preparation of rTCS (**A**) and rTCS-LMWP (**B**). FPLC purification of rTCS (**C**) and rTCS-LMWP (**D**), and SDS-PAGE characterization (**E**). The molecular weight of rTCS (**F**) and rTCS-LMWP (**G**) from MALDI-TOF.

rTCS and rTCS-LMWP were prepared using an IPL (intein-mediated protein ligation) method. The L-cysteine-inducing and intein-mediated cleavage was performed on-column to yield rTCS (**Figure 1A**) or rTCS-LMWP (**Figure 1B**). The collected protein solution was purified via FPLC equipped with a desalting column (**Figure 1C**, **1D**) and characterized by SDS-PAGE electrophoresis (**Figure 1E**) and MALDI-TOF (**Figure 1F**, **1G**).

### Cellular uptake and tumor spheroid penetration of rTCS and rTCS-LMWP

**Figure 2:**
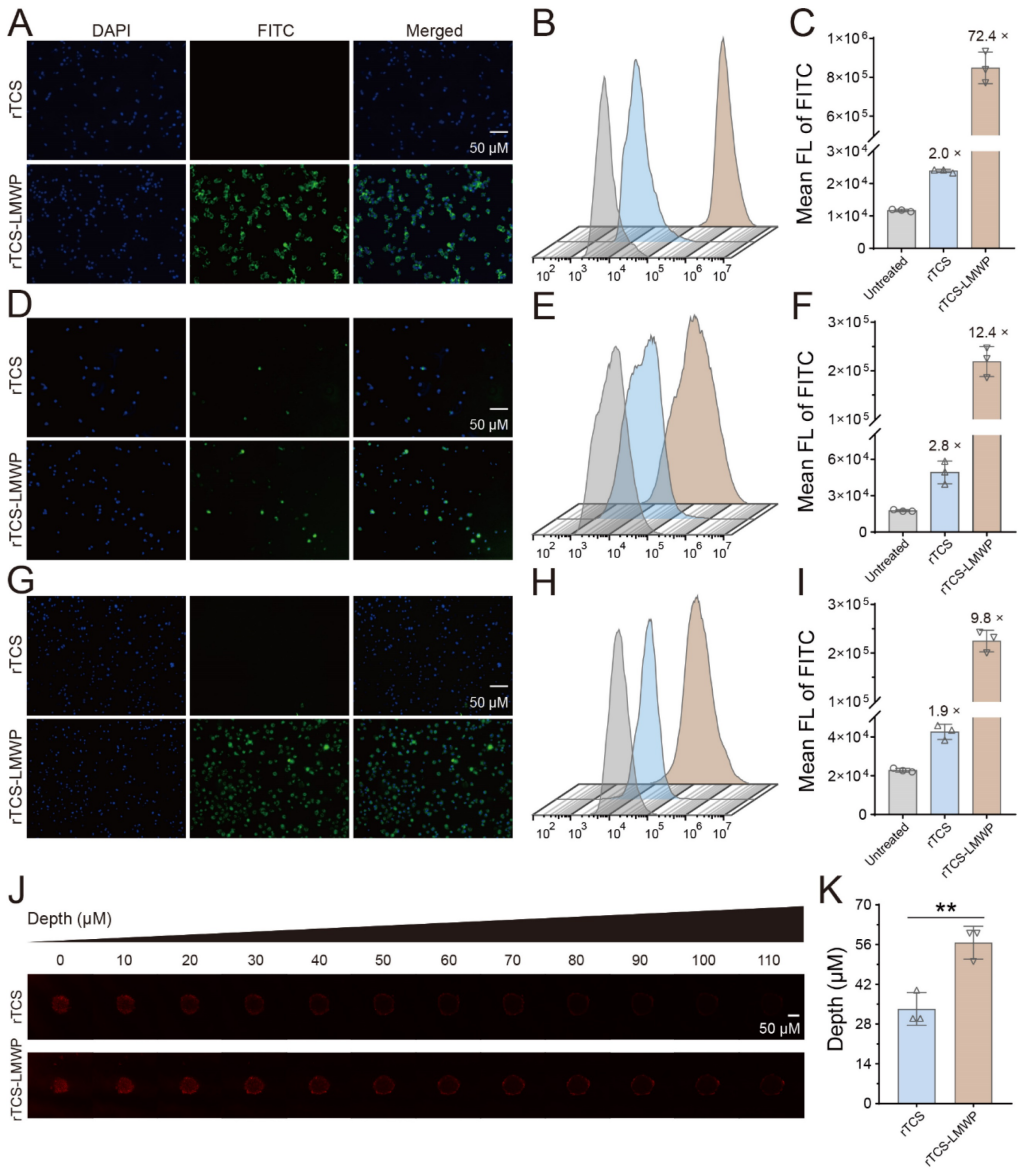
The cellular uptake and tumor spheroid penetration of rTCS and rTCS-LMWP. The cellular uptake of rTCS and rTCS-LMWP in CT26 cells via fluorescence microscope (**A**) and flow cytometry (**B&C**). The cellular uptake of rTCS and rTCS-LMWP in BMDCs via fluorescence microscope (**D**) and flow cytometry (**E&F**). The cellular uptake of rTCS and rTCS-LMWP in M2-type BMDMs via fluorescence microscope (**G**) and flow cytometry (**H&I**). (**J**) CLSM images of CT26 tumor spheroids treated with rTCS and rTCS-LMWP. (**K**) Quantitative analysis of the penetration depth of rTCS and rTCS-LMWP in CT26 tumor spheroids. **p ˂ 0.01.

The enhanced cell/tissue penetration ability of rTCS-LMWP was detected via the cellular uptake assay in CT26, BMDCs, and M2-type BMDMs, and the tumor spheroid penetration assay, with rTCS as a control. The enhanced cellular uptake efficiency of rTCS-LMWP was observed in the cells (**Figure 2A-I**). Furthermore, rTCS-LMWP exhibited enhanced tumor spheroid penetration compared to rTCS (**Figure 2J**,** 2K**). It indicated that LMWP promoted the intracellular delivery and tissue penetration of the protein.

Besides, the *in vitro* biocompatibility of rTCS-LMWP on BMDCs, M2-like BMDMs, and normal cells such as bEnd3 and HUVEC was detected via MTT assay. The cell viability was all above 70% after treatment with 5 μM of rTCS-LMWP for 24 h (**Figure S1**, **Supporting Information**).

### Immunogenic cell death (ICD) effect

ICD is a specific regulatory cell death that is driven by stress and induces adaptive immunity against the antigens released from the dying cells 25. The ICD-occurring cancer cells release the ATP and high-mobility group box 1 protein (HMGB1) and expose calreticulin (CRT) to the serosal surface 26. The extracellular ATP sends a “finding-me” signal to DC progenitors and macrophages, thereby promoting the recruitment of myeloid cells to the tumor; HMGB1 binds multiple pattern recognition receptors expressed by myeloid cells to activate immunity; the exposed CRT serves as an “eating-me” signal and promotes the phagocytosis of tumor cells by phagocytes 27. ICD plays an important role in triggering an anti-tumor immune response.

**Figure 3:**
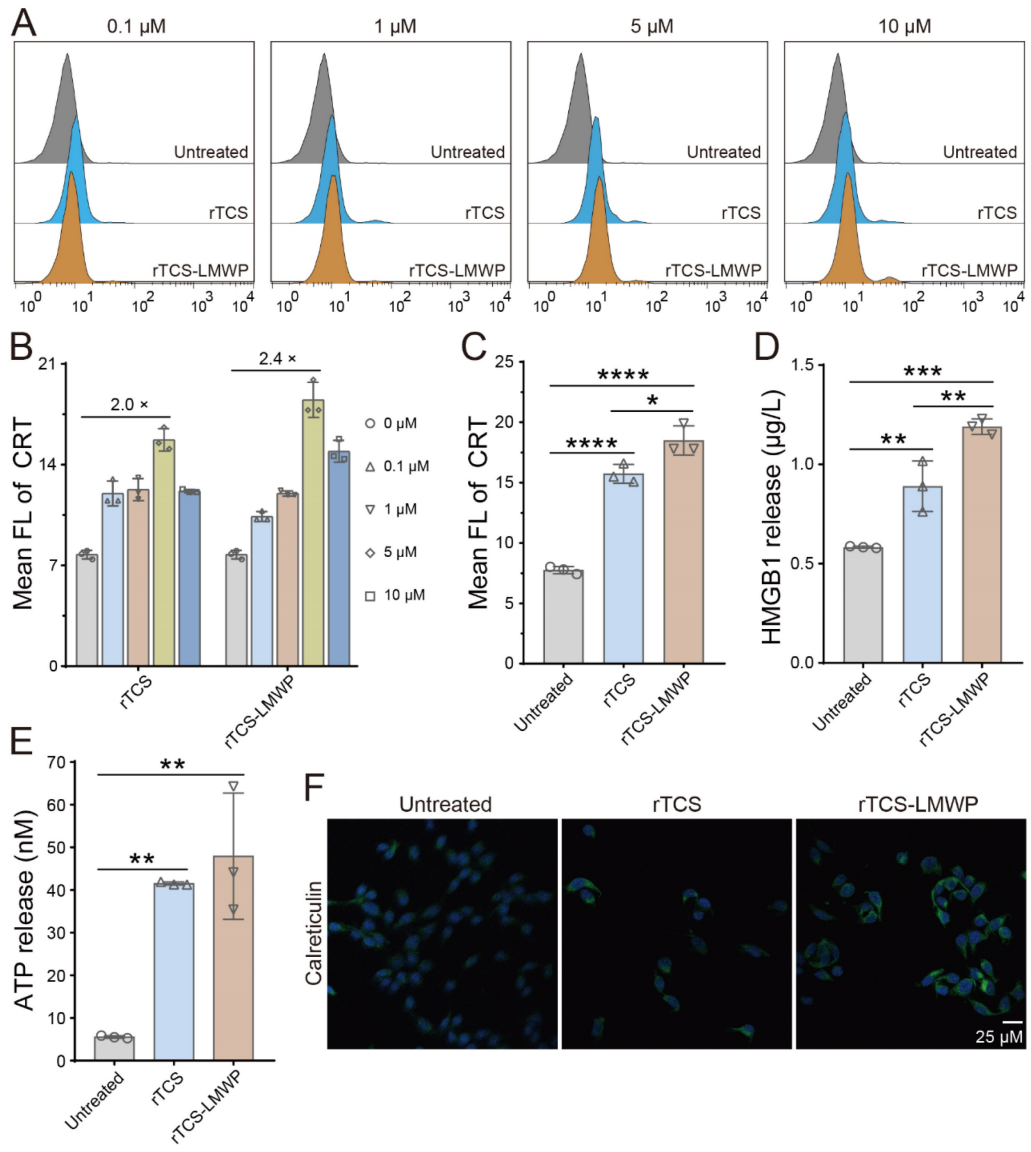
ICD induction of rTCS and rTCS-LMWP on CT26 cells. (**A**) Calreticulin (CRT) exposure of CT26 treated with rTCS and rTCS-LMWP by flow cytometry. (**B**) Statistical analysis of the mean fluorescence intensity in (**A**). (**C**) Mean fluorescence intensity of CRT of CT26 after treatment with 5 µM of rTCS and rTCS-LMWP. (**D**) HMGB1 level in the tumor cell culture supernatant after rTCS and rTCS-LMWP treatment. (**E**) ATP level in the tumor cell culture supernatant after rTCS and rTCS-LMWP treatment. (**F**) CLSM images of calreticulin of CT26 treated with rTCS and rTCS-LMWP. *p ˂ 0.05; **p ˂ 0.01; ***p ˂ 0.001; ****p ˂ 0.0001.

The ICD induction of rTCS-LMWP was demonstrated by the results of flow cytometry and immunofluorescence staining. The CRT level was increased after treatment with rTCS-LMWP (**Figure 3A**), in a dose-dependent manner at a concentration range of 0.1-5 μΜ. At a higher concentration (10 μΜ), the tumor cells can be directly killed because of the enhanced cytotoxic effect of TCS, and the ICD induction was weakened. The optimal effect was achieved at a concentration of 5 μΜ, and rTCS-LMWP showed higher efficacy than rTCS (**Figure 3B**, **3C**). The results were also confirmed by the immunofluorescence staining of CRT (**Figure 3F**). Correspondingly, the level of HMGB1 and ATP released from the tumor cells was increased significantly after rTCS-LMWP treatment (**Figure 3D**, **3E**).

### BMDC and BMDM stimulation of rTCS-LMWP

**Figure 4:**
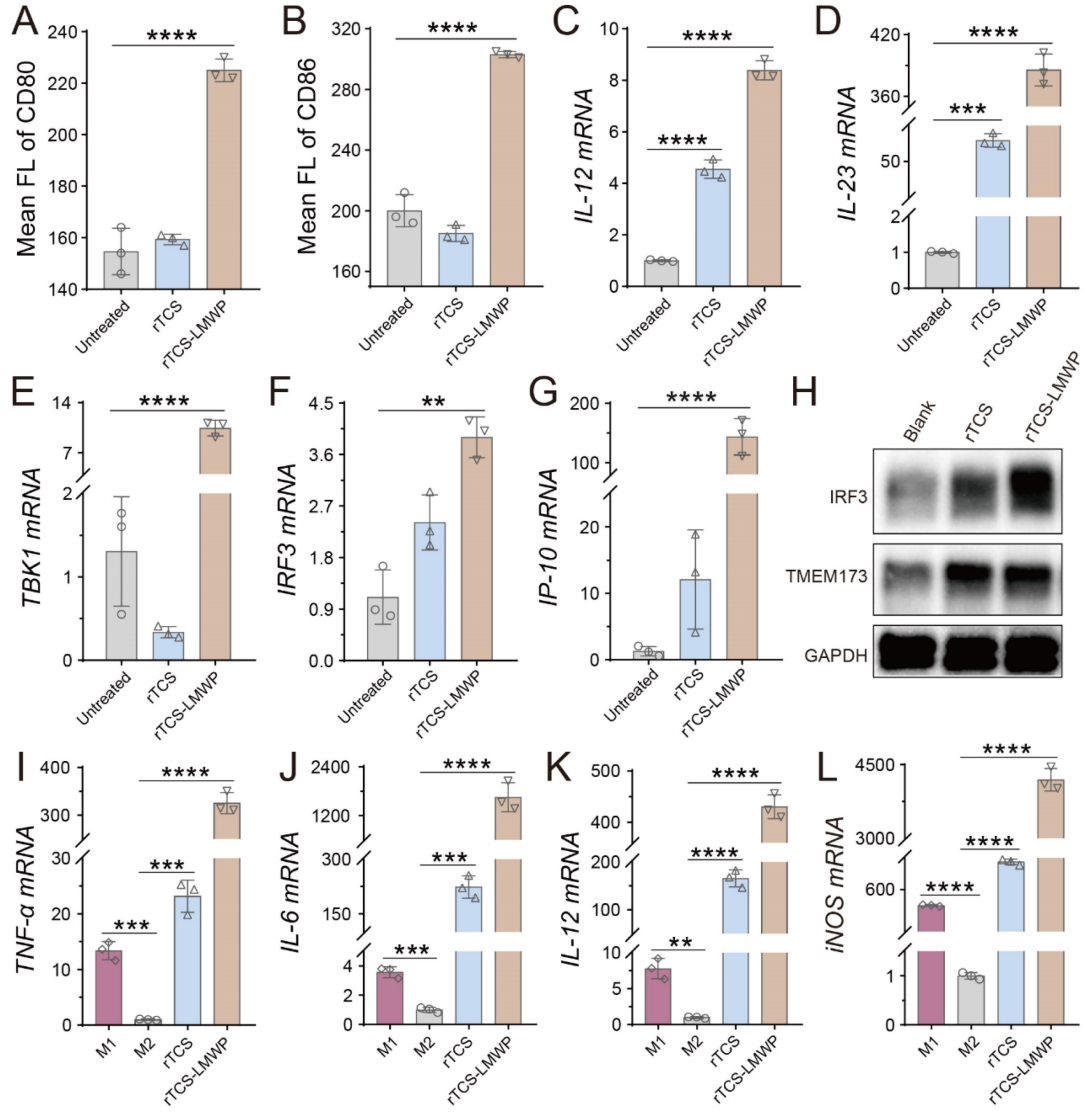
The stimulating effect of rTCS and rTCS-LMWP on BMDCs and BMDMs. The mean fluorescence intensity of CD80 (**A**) and CD86 (**B**) of BMDCs after treatment. The mRNA levels of IL-12 (**C**), IL-23 (**D**), TBK1 (**E**), IRF3 (**F**), and IP-10 (**G**) of BMDCs after treatment. (**H**) The protein expression of IRF3 and TMEM173 (STING) of BMDCs measured by western blotting. (**I-L**) The mRNA levels of TNF-α, IL-6, IL-12, and iNOS of BMDMs treated with rTCS and rTCS-LMWP. **p ˂ 0.01; ***p ˂ 0.001; ****p ˂ 0.0001.

Dendritic cells (DCs) are professional antigen-presenting cells (APCs) and play key roles in initiating, regulating, and maintaining the anti-tumor immune response. The mature DCs facilitate the anti-tumor immune response, while the immature DCs lead to an impotent T-cell immune response due to lacking the necessary co-stimulators for activating T cells 28, 29. The matured DCs are characterized by upregulating the co-stimulators (e.g., CD80 and CD86) and MHC-II, and expressing the cytokines essential for priming T-cell immune responses, such as IL-12 and IL-23 28, 30. Our results showed that the DCs treated with rTCS-LMWP exhibited higher expression of CD80 and CD86 (**Figure 4A**, **4B**), as well as MHC-II (**Figure S2A**), compared with rTCS that had a low cell penetration ability. The mRNA levels of IL-12 and IL-23 were also significantly upregulated after rTCS-LMWP treatment (**Figure 4C**, **4D**). Other pro-inflammatory molecules, like IL-1β and IL-6, were also upregulated (**Figure S2B**, **S2C**), while the anti-inflammatory IL-10 had no obvious change (**Figure S2D**), further demonstrating the activation effect of rTCS-LMWP on BMDCs.

The stimulator of interferon genes (STING) pathway is activated by the cytoplasmic DNA released from infectious microorganisms or damaged autologous cells and thus initiates the immune responses 31. The STING subsequently activates the TBK1-IRF3 signal pathway and induces the type I IFN expression, resulting in activated innate immune responses 32. The related molecules of the STING pathway, such as STING (aka TMEM173), TBK1, and IRF3, were detected. The results showed that TBK1 and IRF3 mRNA was significantly upregulated in the BMDCs treated with rTCS-LMWP (**Figure 4E**, **4F**), and the downstream interferon-inducible protein 10 (IP-10) was also increased (**Figure 4G**). The increased protein expression of IRF3 and TMEM173 was observed via western blotting assay (**Figure 4H**). These results revealed that rTCS-LMWP treatment could activate the STING pathway to stimulate BMDCs.

Macrophages are potent phagocytic cells and can also serve as APCs to present antigens to promote an adaptive immune response 33. However, tumor-associated macrophages (TAMs) are primarily the pro-tumor phenotype (M2) in TME, instead of the anti-tumor phenotype (M1). Therefore, the repolarization of TAMs from M2 to M1 can effectively mitigate the tumor immunosuppressive microenvironment (TIME) and activate anticancer immunity 19, 34, 35. After rTCS-LMWP treatment, the M2 BMDMs were repolarized toward the M1 phenotype, as reflected by the increased levels of the M1-related mRNA (e.g., TNF-α, IL-6, IL-12, and iNOS) (**Figure 4I-L**).

### *In vivo* therapeutic effect

**Figure 5:**
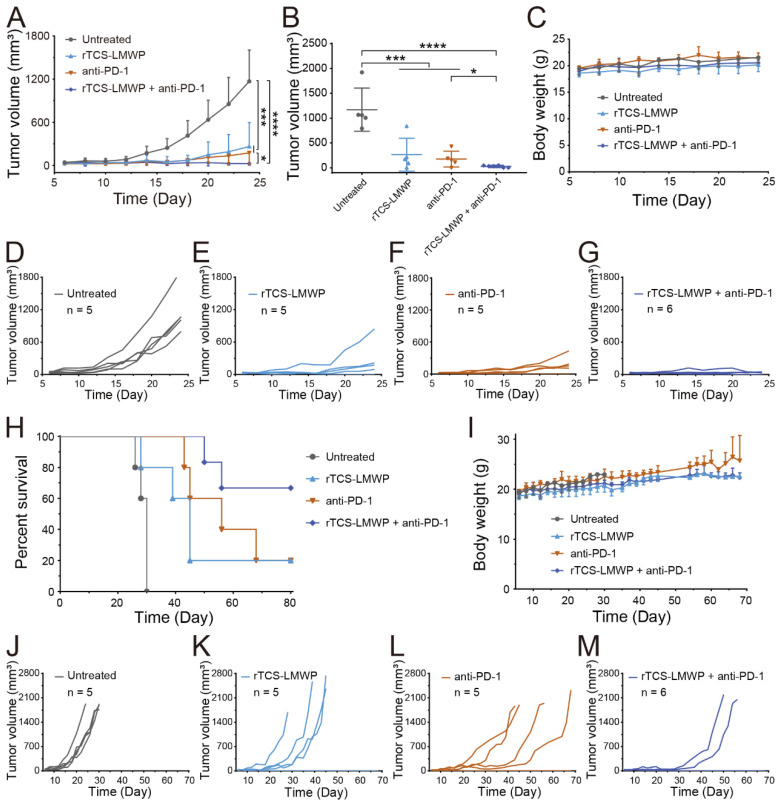
The synergistic anti-tumor effect and survival study of rTCS-LMWP combined with anti-PD-1 on CT26 subcutaneous tumor model. (**A**) The tumor growth curve during 24-day treatment. (**B**) The tumor volume on the 24^th^ day. (**C**) The body weight during 24-day treatment. The individual tumor growth curve of the untreated group (**D**), rTCS-LMWP group (**E**), anti-PD-1 group (**F**), and rTCS-LMWP + anti-PD-1 group (**G**) during 24-day treatment. (**H**) The survival curve of mice. (**I**) The body weight changes. The individual tumor growth curve of the untreated group (**J**), rTCS-LMWP group (**K**), anti-PD-1 group (**L**), and rTCS-LMWP + anti-PD-1 group (**M**). *p ˂ 0.05; ***p ˂ 0.001; ****p ˂ 0.0001.

The synergistic effect of rTCS-LMWP in combination with anti-PD-1 therapy was investigated in a CT26 subcutaneous tumor model. The tumor growth in the untreated group was very rapid after inoculation. By contrast, the combo of rTCS-LMWP and anti-PD-1 exhibited potent antitumor activity (**Figure 5A-G**), with much higher efficacy than the single treatment of either rTCS-LMWP or anti-PD-1.

On the 28^th^ day after tumor inoculation, no mice in the untreated group survived. On the 68^th^ day, merely 20% of mice survived in the single treatment group (rTCS-LMWP or anti-PD-1). By contrast, 80% of mice in the combo group remained, showing a significantly prolonged survival rate (**Figure 5H-M**). These results demonstrated the synergistic effect of the combination therapy of rTCS-LMWP and anti-PD-1.

### *In vivo* remodeling of the TIME

**Figure 6:**
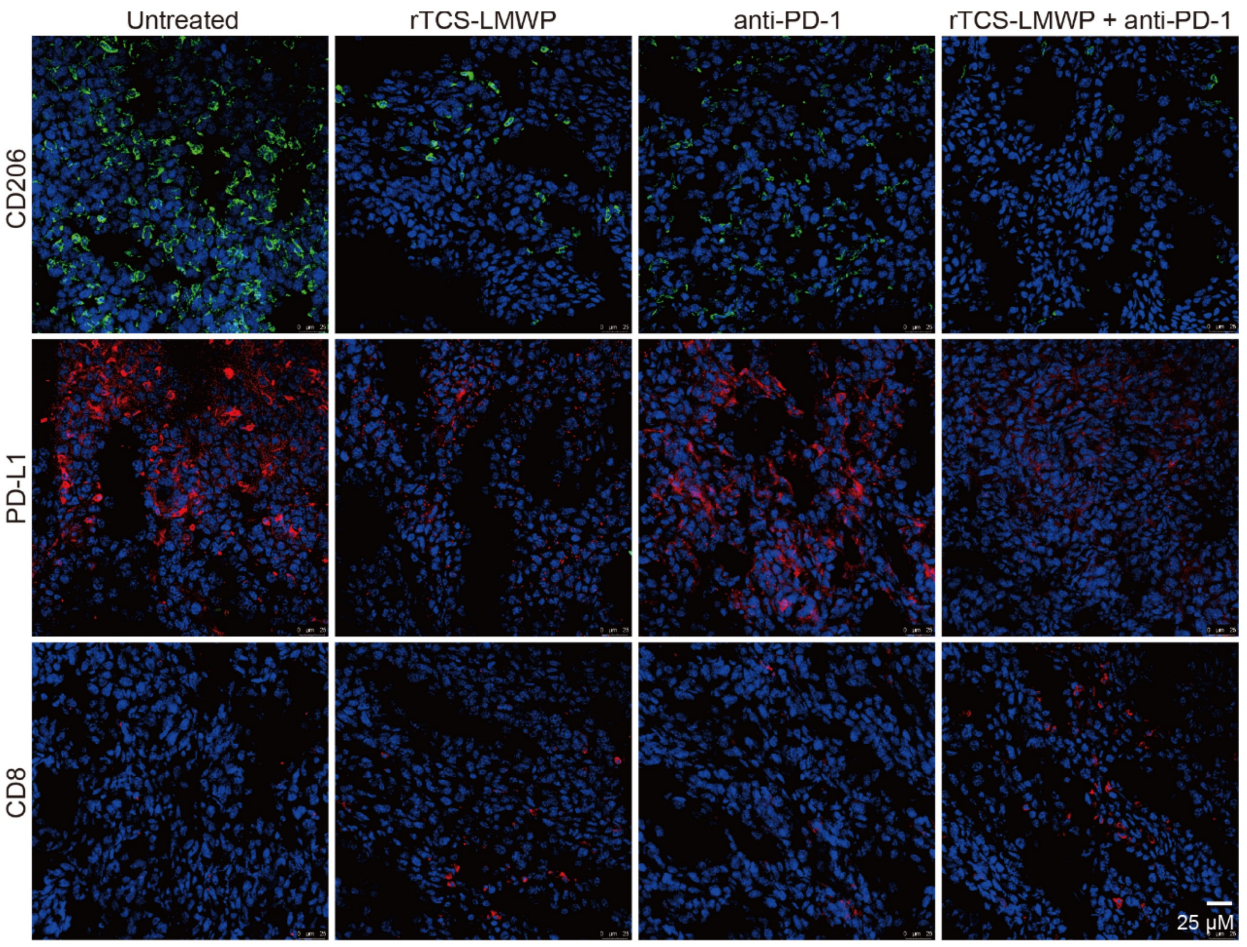
The immunofluorescent staining of CD206, PD-L1, and CD8 in the tumor tissues at the treatment endpoint.

CD206^+^, PD-L1^+^, and CD8^+^ T cells in the tumor tissues after the therapeutic experiment were detected by immunofluorescence staining. The results showed that the intratumoral infiltration of CD8^+^ T cells was significantly increased in the combination therapy group containing anti-PD-1; in addition, the combination therapy significantly down-regulated the levels of PD-L1 and CD206 in the tumor (**Figure 6**). Notably, PD-L1 and CD206 are overexpressed in the M2-type TAMs 36, 37. Therefore, the results suggested the repolarization of M2-type TAMs.

**Figure 7:**
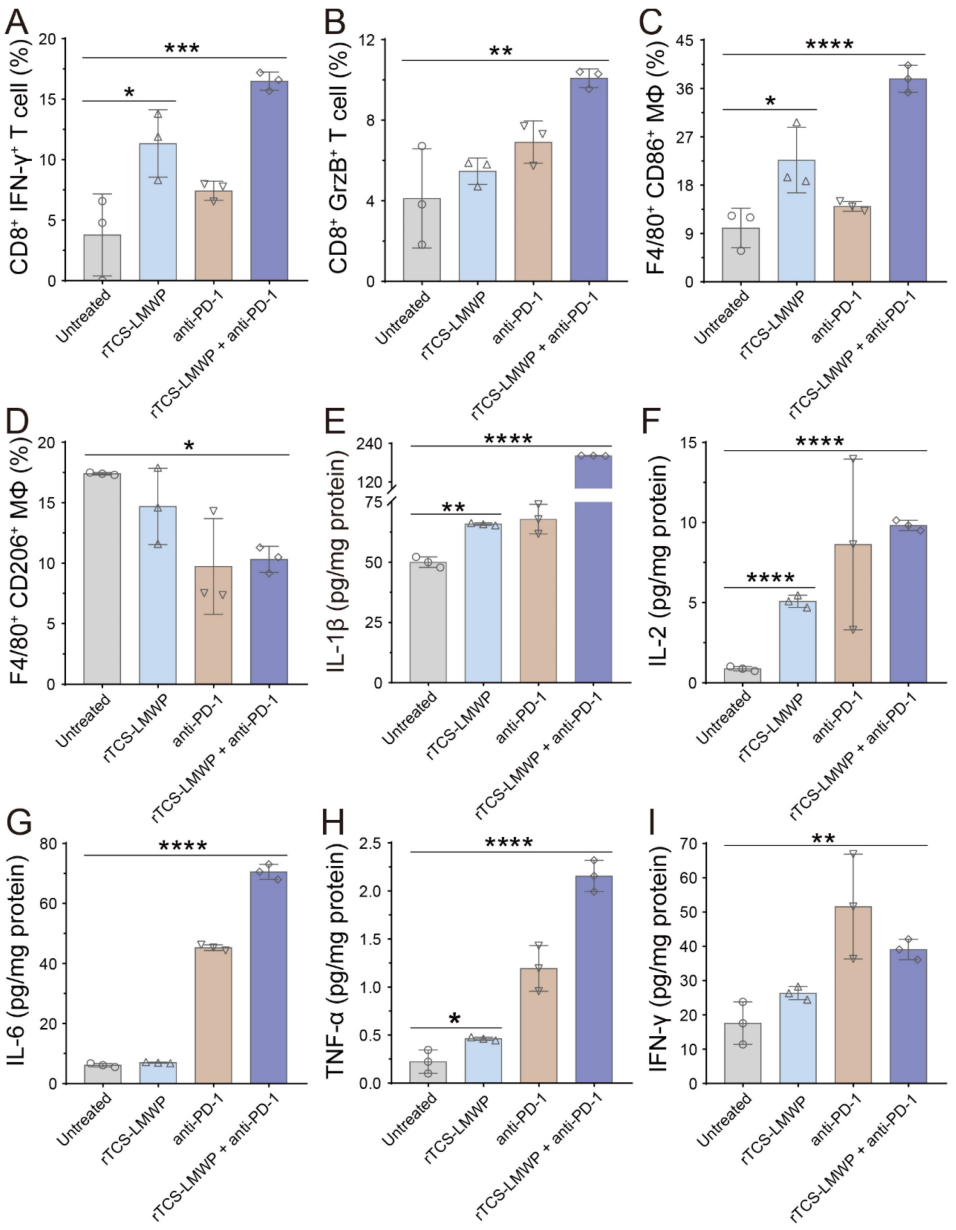
The *in vivo* remodeling of TIME on the 7^th^ day after the last treatment. (**A**) The population of CD8^+^ IFN-γ^+^ T cells in the tumor tissues. (**B**) The population of CD8^+^ GrzB^+^ T cells in the tumor tissues. (**C**) The population of F4/80^+^ CD86^+^ macrophages in the tumor tissues. (**D**) The population of F4/80^+^ CD206^+^ macrophages in the tumor tissues. (**E-I**) The intratumoral level of IL-1β, IL-2, IL-6, TNF-α, and IFN-γ. *p ˂ 0.05; **p ˂ 0.01; ***p ˂ 0.001; ****p ˂ 0.0001.

For a further study of the TIME remodeling, the tumor tissues were collected for detecting the intratumoral immune cells and cytokines via flow cytometry and ELISA assay. In the rTCS-LMWP group and rTCS-LMWP + anti-PD-1 group, the percentages of CD8^+^ Ki67^+^ T cells, CD8^+^ IFN-γ^+^ T cells, and CD8^+^ Granzyme B^+^ T cells were increased, demonstrating the enhanced T cell immunity (**Figure 7A&B**, **Figure S3A&B**, and **Figure S4A&B**). The combination therapy led to the increased population of F4/80^+^ CD86^+^ macrophages (M1-type) and the decreased population of F4/80^+^ CD206^+^ macrophages (M2-type), indicating the M2→M1 repolarization of TAMs (**Figure 7C&D**, **Figure S3C&D**). Meanwhile, the combination group showed an increased intratumoral F4/80^+^ CD169^+^ population of macrophages, illustrating the enhanced antigen-presenting ability of macrophages (**Figure S4G&H**). Furthermore, the intratumoral CD4^+^ T helper cells were also increased (**Figure S4C&D**), whereas the immunosuppressive regulatory T cells (Tregs, CD4^+^ FoxP3^+^) were reduced (**Figure S4E&F**), implying the alleviated immunosuppression of TME.

TME is characterized by the hypersecretion of anti-inflammatory cytokines for escaping immune surveillance 38. The intratumoral levels of the anticancer cytokines (e.g., IL-1β, IL-2, IL-6, TNF-α, and IFN-γ) in the treated groups were all increased, and the combination therapy showed the highest increase (**Figure 7E-I**). These results indicated that rTCS-LMWP enhanced anticancer immunity and assisted anti-PD-1 therapy.

Moreover, the increased CD8^+^ Ki67^+^ T cells and MHC-II expression of CD11c^+^ DCs after combination treatment suggested the proliferation of CD8^+^ T cells and activation of DCs in the tumor-draining lymph nodes (**Figure S5A-D**).

**Figure 8:**
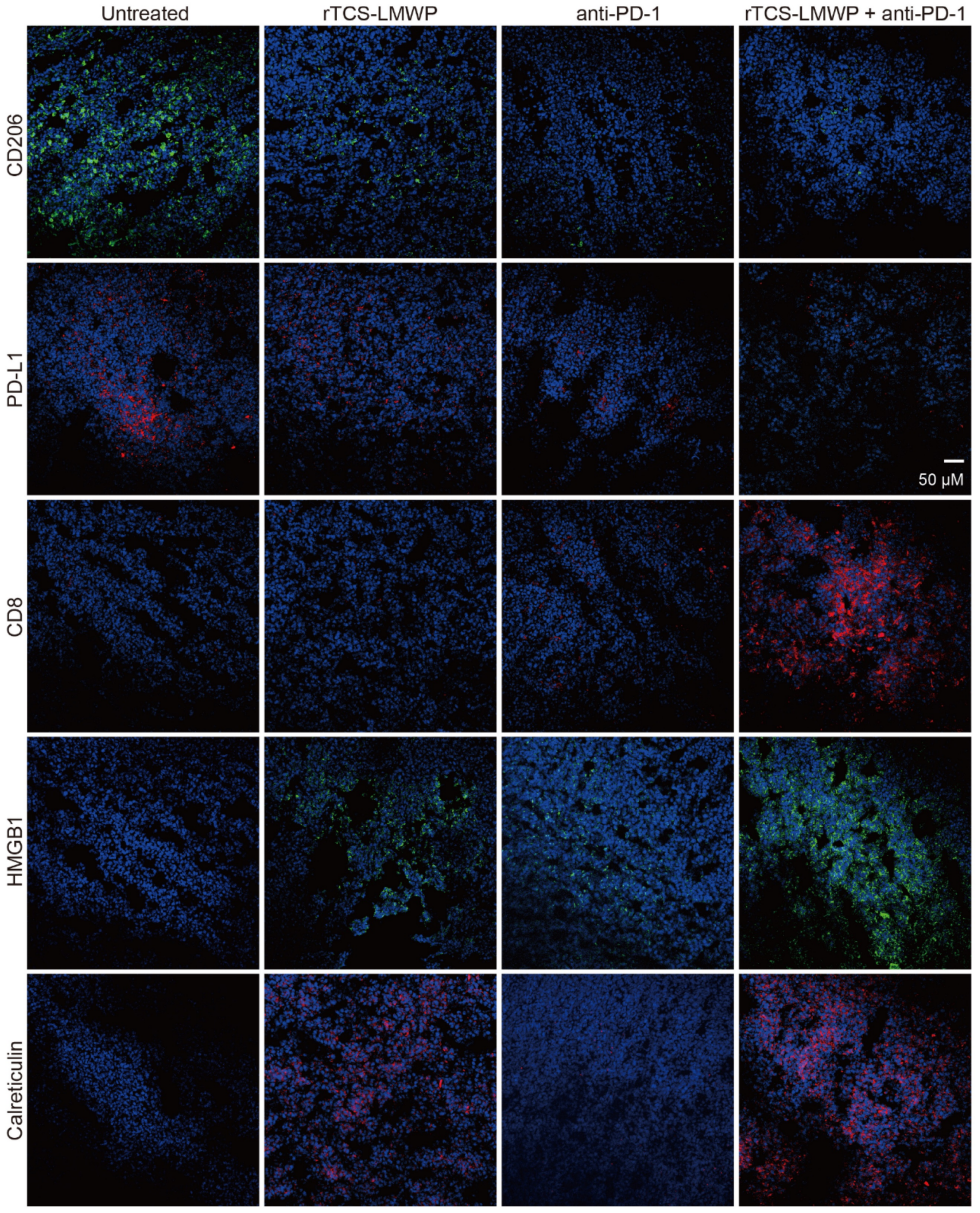
The immunofluorescence staining of CD206, PD-L1, CD8, HMGB1, and calreticulin of tumor tissues on the 7^th^ day after the last treatment.

The immunofluorescence staining of the tumor tissues also illustrated the ameliorated TIME by combination therapy. The results showed that the pro-tumor biomarkers of CD206 and PD-L1 were downregulated, but the CD8^+^ cytotoxic T cell increased (**Figure 8**). HMGB1 and calreticulin were also upregulated in the tumors after combination therapy, demonstrating the ICD induction (**Figure 8**).

In summary, the results above demonstrated the remodeling of the tumor immune microenvironment and efficiently activated anticancer immunity.

### *In vivo* biosafety of rTCS-LMWP

The preliminary safety of rTCS-LMWP was tested. All the mice had no significant change in body weight (**Figure S6A**). The rTCS-LMWP-treated mice showed no significance in organ coefficients compared to the untreated group (**Figure S6B**). Notably, rTCS-LMWP treatment did not cause significant changes in spleen enlargement (**Figure S6B**) and serum inflammatory cytokines (**Figure S6C**), indicating the low possibility of rTCS-LMWP causing immune-related adverse events (irAE). Besides, the H&E staining of the injection-site skin showed no observed inflammatory response after treatment with rTCS-LMWP (**Figure S6D**).

## Discussion

The clinical application of anti-PD-1/PD-L1 has been limited by the low response rate 39. TME plays a critical role in determining the response rate to anti-PD-1/PD-L1 therapy. In the TME, the tumor cells have low tumor antigen expression and the immune cells are in a dysfunctional state, resulting in poor tumor-antigen recognition and presentation, further leading to poor tumor-specific T-cell responses 38. Therefore, increasing tumor antigenicity and activating antigen-presenting cells are essential for improving anti-PD-1/PD-L1 therapy.

In our previous work, TCS was applied as a vaccine adjuvant and successfully induced cancer immunization 18, 19. But its application as an adjuvant therapy to the mainstay immune checkpoint therapy was not yet revealed. In this work, a recombinant TCS derivative (rTCS-LMWP) with cell-penetrating ability was prepared and its immunomodulatory effect on synergizing anti-PD-1 therapy was demonstrated. rTCS-LMWP can induce tumor cells to occur ICD, thereby causing the release of “danger” signaling molecules to stimulate DCs. Meanwhile, rTCS-LMWP can promote DC maturation by activating the STING-TBK1-IRF3 axis, and also repolarize the macrophages toward a pro-inflammatory phenotype, thus enhancing the vigilance function of APCs. Therefore, the combination of rTCS-LMWP and anti-PD-1 can significantly enhance immunotherapy efficacy. The remodeling of TIME was reflected by the induction of ICD, the enhanced proliferation and functionalization of cytotoxic T cells, and the M2→M1 repolarization of macrophages.

To seek a safe and effective immunostimulant for assisting immunotherapy is an urgent but unmet need. The rTCS-LMWP can effectively inhibit tumor growth and prolong the overall survival of mice at a low intratumoral dose, and alleviate the tumor immunosuppression to synergize the anti-PD-1 therapy with no detectable effect on the systemic immune response.

There are some immune activators such as CpG ODNs and poly(I:C), which are often investigated in preclinical studies. However, their clinical translation is still limited and long-term safety needs further investigation 40. Notably, apart from the immunostimulation effect, CpG was reported to simultaneously induce the compensatory expression of immunosuppressive molecules that compromised the anti-PD-1 efficacy 41. Trichosanthin is an approved drug in China and has been clinically used for over 3 decades. The drug reposition of trichosanthin is a potential application for cancer immunotherapy because it would be cost- and time-saving and benefit from the well-documented safety of TCS from the decade-long clinical practice. However, the native TCS protein is not favorable for application as an anticancer drug due to its poor intratumor diffusion and cell permeability. The recombinant rTCS-LMWP can improve delivery efficiency, with the benefit of an easy preparation process at a low cost.

## Conclusion

In this work, the recombinant cell-penetrating rTCS-LMWP has been constructed and it can serve as an adjuvant to anti-PD-1 therapy. rTCS-LMWP can remodel the tumor immune microenvironment by inducing the ICD of tumor cells, stimulating DCs, repolarizing TAMs, increasing cytotoxic T cells, and suppressing Tregs, thus assisting anti-PD-1 therapy. It indicates the promise of the translation of such an old drug into new applications as a novel immunomodulator to synergize the anti-PD-1/PD-L1 therapy. Therefore, rTCS-LMWP is of potential clinical value.

## Supplementary Material

Supplementary figures and table.
