# Supplementary material for: Recombinant cell-penetrating trichosanthin synergizes anti-PD-1 therapy in colorectal tumor
Source: Int J Biol Sci. 2023 Mar 5;19(6):1698–712. doi: 10.7150/ijbs.81052 (PMC10092770; doi:10.7150/ijbs.81052)

Supporting Information

Recombinant cell-penetrating trichosanthin synergizes anti-PD-1 therapy in colorectal tumor

Table S1 The primers for RT-qPCR.

Target Gene	Forward sequence (5' to 3')	Reverse sequence (5' to 3')
Mus GAPDH	AGGTCGGTGTGAACGGATTTG	TGTAGACCATGTAGTTGAGGTCA
Mus IL-1β	CTTCAGGCAGGCAGTATCACTC	TGCAGTTGTCTAATGGGAACGT
Mus IL-6	GTCTGTAGCTCATTCTGCTCTG	GAAGGCAACTGGATGGAAGT
Mus IL-10	TTTCAAACAAAGGACCAG	GGATCATTTCCGATAAGG
Mus IL-12	AGACATCACACGGGACCAAAC	CCAGGCAACTCTCGTTCTTGT
Mus IL-23	TAATGCTATGGCTGTTGCCCT	TTCATATGTCCCGCTGGTGC
Mus TBK1	GGAGCCGTCCAATGCGTAT	GCCGTTCTCTCGGAGATGATTC
Mus IRF3	GAGAGCCGAACGAGGTTCAG	CTTCCAGGTTGACACGTCCG
Mus IP-10	CCAAGTGCTGCCGTCATTTTC	GGCTCGCAGGGATGATTTCAA
Mus TNF-α	CCCTCACACTCAGATCATCTTCT	GCTACGACGTGGGCTACAG
Mus iNOS	CCCTTCAATGGTTGGTACATGG	ACATTGATCTCCGTGACAGCC

Figure S1 The in vitro biocompatibility of rTCS and rTCS-LMWP. The cell viability of BMDCs (**A**), M2-type BMDMs (**B**), bEnd3 (**C**), and HUVEC (**D**) after treatment with different concentrations of rTCS and rTCS-LMWP.

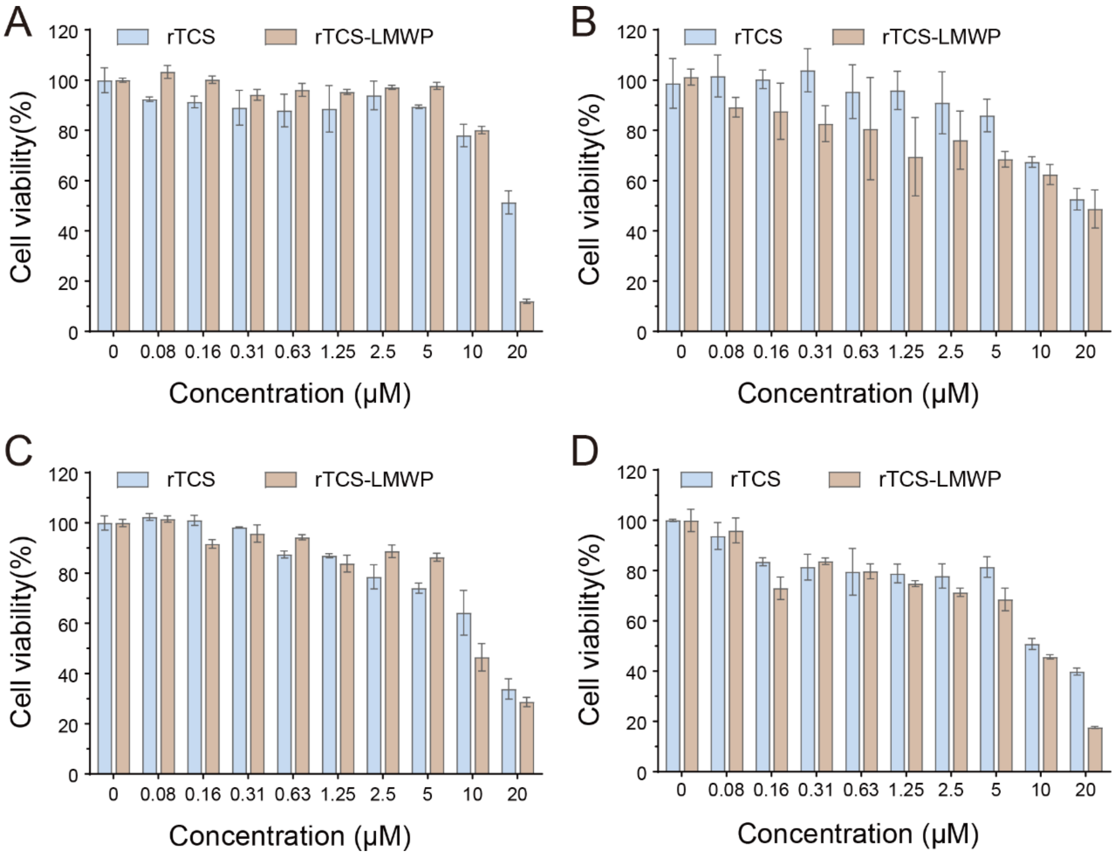

Figure S2 The stimulating effect of rTCS and rTCS-LMWP on BMDCs. (**A**) The mean fluorescence of MHC-II of BMDCs treated with rTCS and rTCS-LMWP by flow cytometry. The mRNA level of IL-1β (**B**), IL-6 (**C**), and IL-10 (**D**) of BMDCs treated with rTCS and rTCS-LMWP by RT-qPCR. **p < 0.01; ***p < 0.001; ****p < 0.0001; ns, not significant.

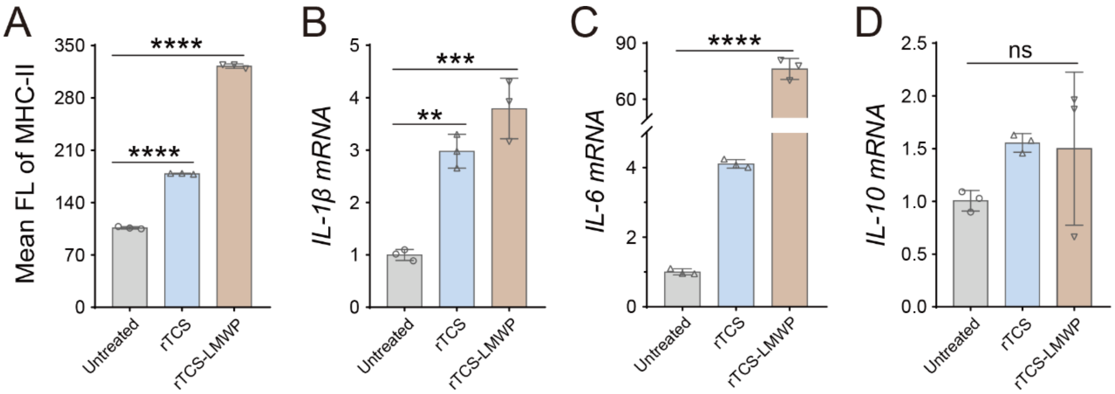

Figure S3 The in vivo remodeling of TIME on the 7th day after the last treatment. The intratumoral population of CD8+ IFN-γ+ T cells (**A**), CD8+ GrzB+ T cells (**B**), F4/80+ CD86+ macrophages (**C**), and F4/80+ CD206+ macrophages (**D**).

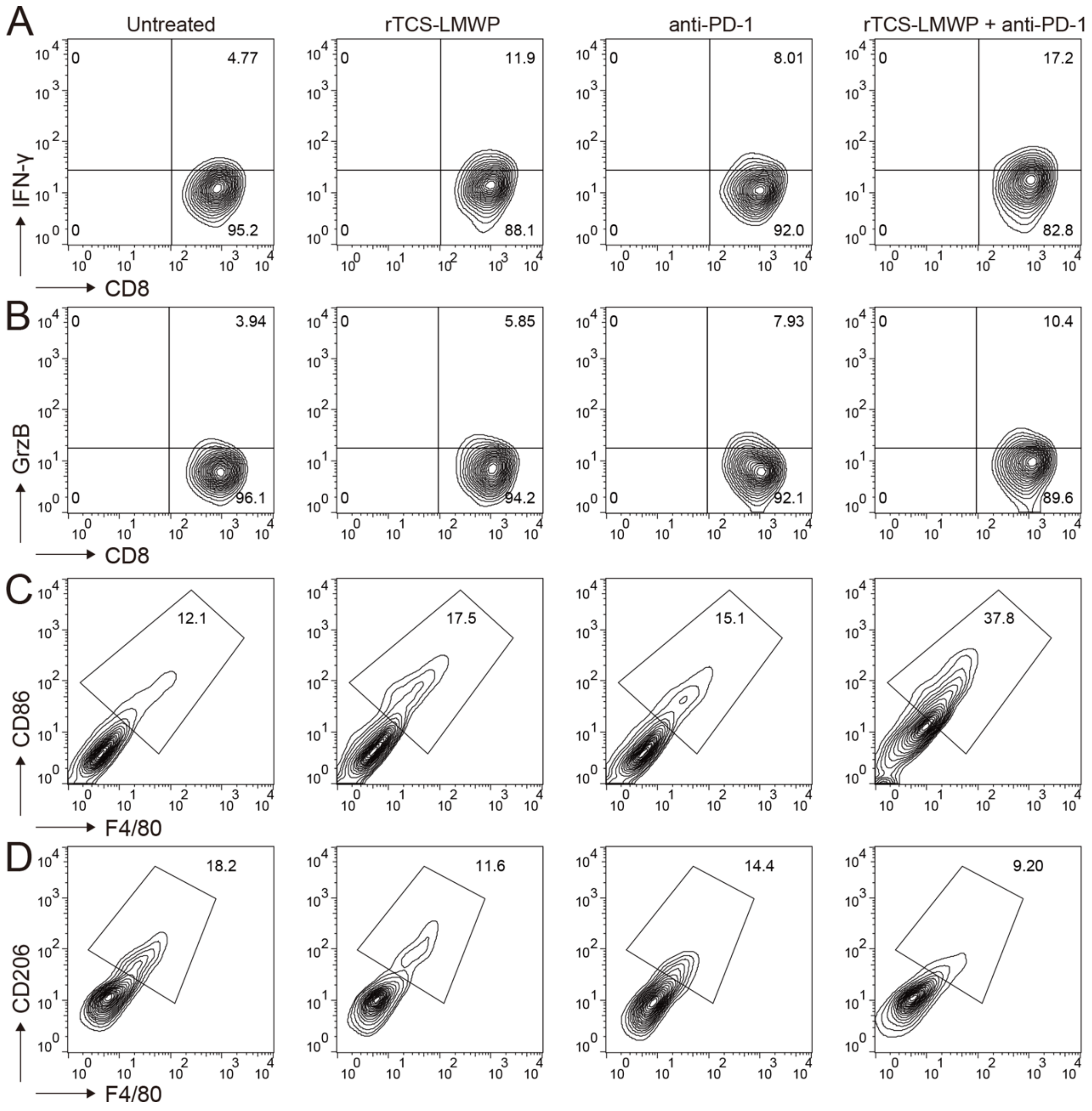

Figure S4 The in vivo immune regulation on the 7th day after the last treatment. (**A&B**) The population of CD8+ Ki67+ T cells in the tumor tissues. (**C&D**) The population of CD4+ T cells in the tumor tissues. (**E&F**) The population of CD4+ FoxP3+ T cells in the tumor tissues. (**G&H**) The population of F4/80+ CD169+ macrophages in the tumor tissues. *p < 0.05; **p < 0.01; ****p < 0.0001.

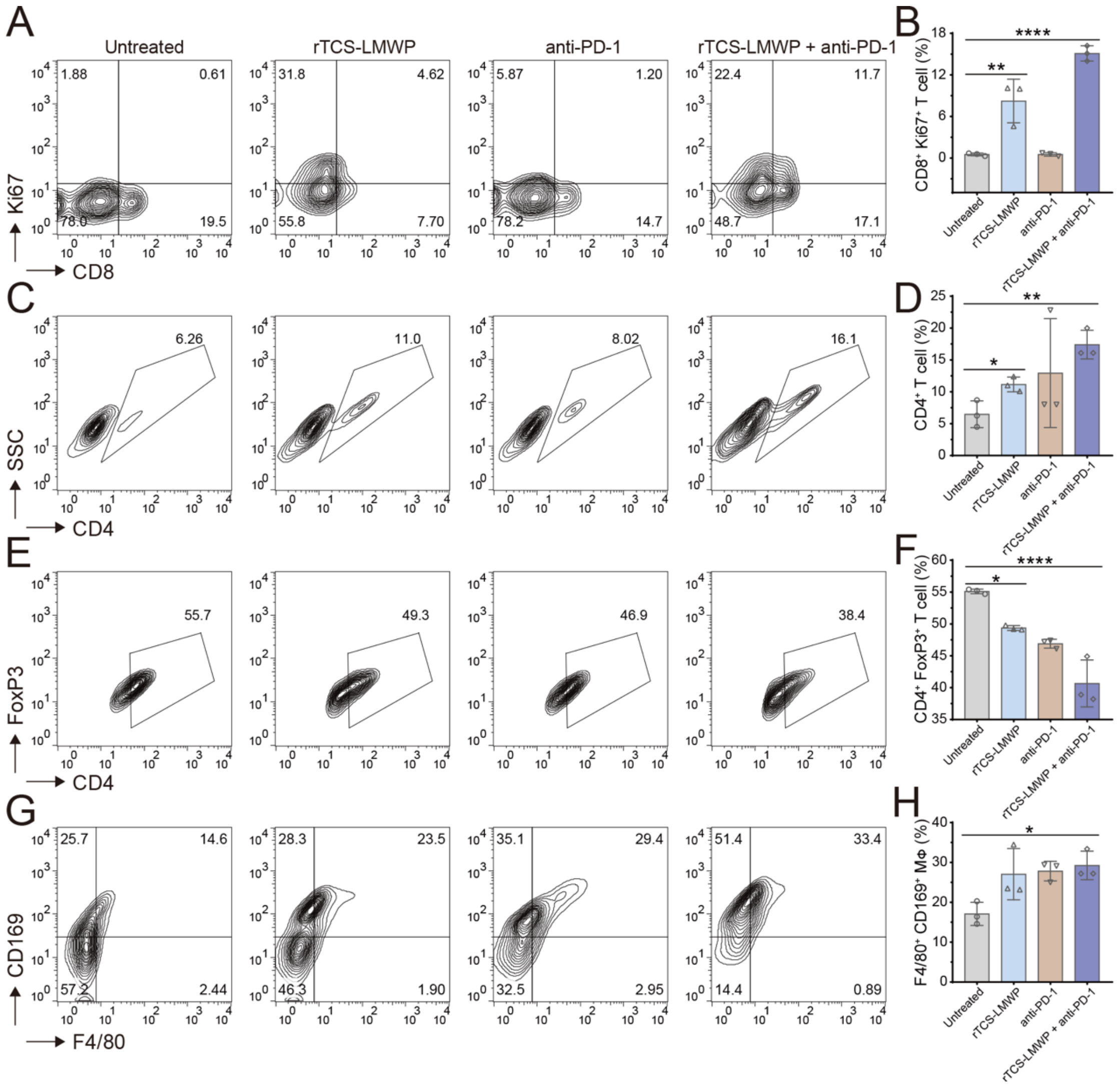

Figure S5 The in vivo immune regulation on the 7th day after the last treatment. (**A&C**) The population of CD8+ Ki67+ T cells in the tumor-draining lymph node. (**B&D**) The mean fluorescence intensity of MHC-II of CD11c+ cells in the tumor-draining lymph node. **p < 0.01; ****p < 0.0001.

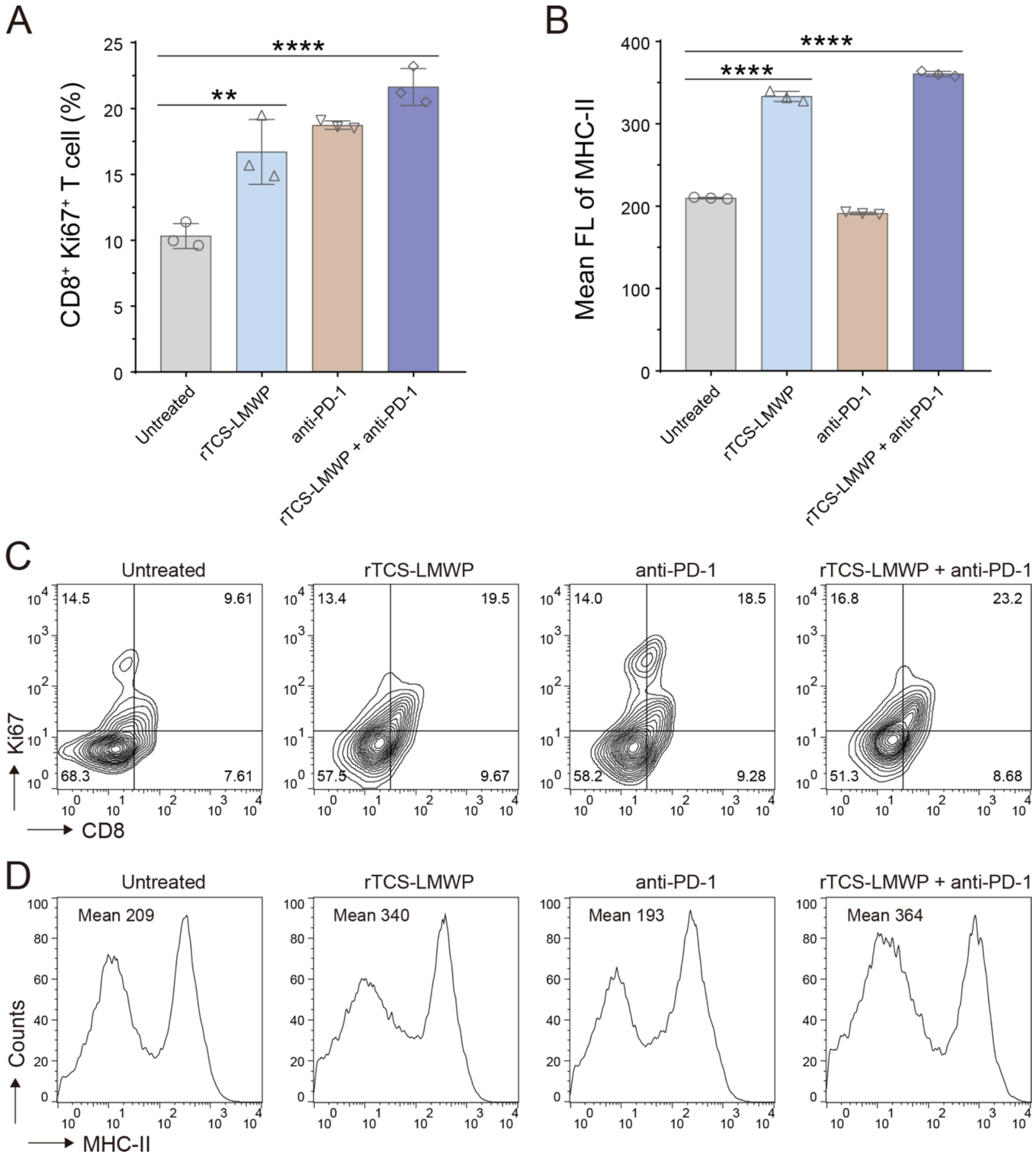

Figure S6 The preliminary in vivo biosafety of rTCS-LMWP in BALB/c mice. (**A**) The body weight. (**B**) The organ coefficient. (**C**) The levels of serum IL-6 and TNF-α. (**D**) The H&E staining of the skin tissue at the injected sites.

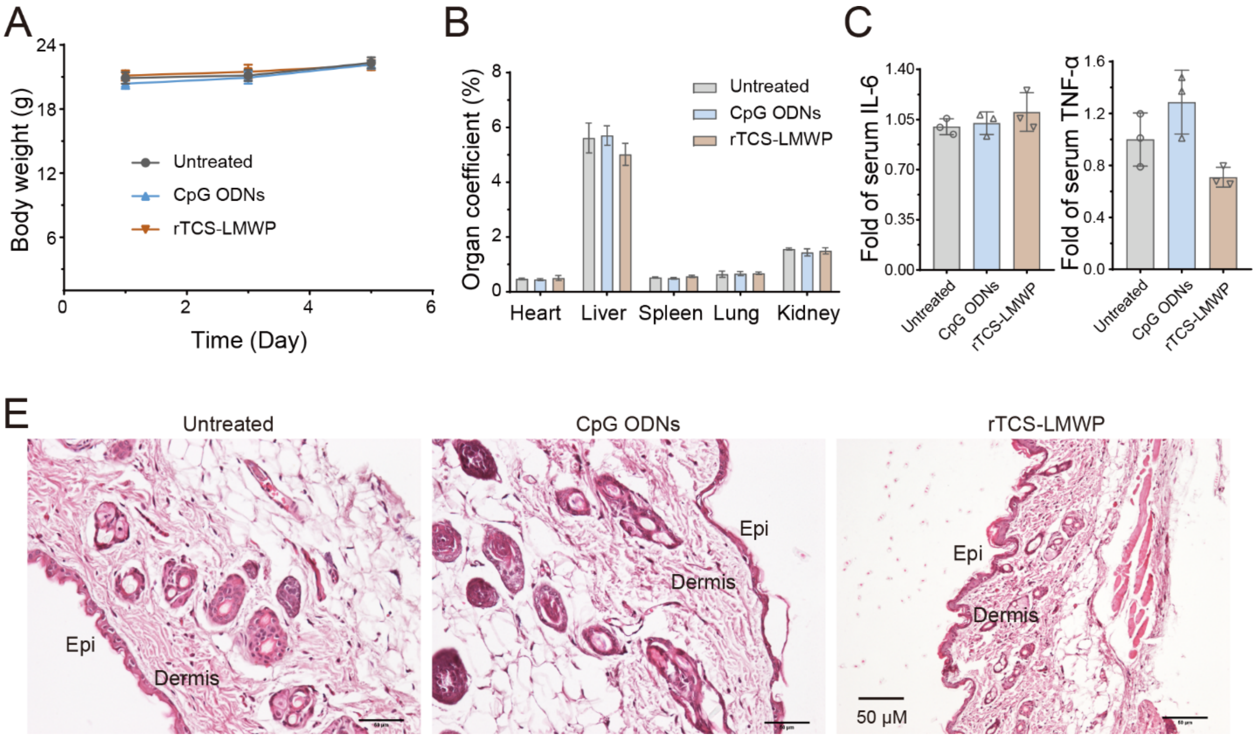

Supplement: Supplementary file 1 — Supplementary figures and table. [file ijbsv19p1698s1.pdf]
